# NGR1 Ameliorates Hepatocyte Steatosis and Mitochondrial Dysfunction Associated with the Restoration of NDUFS2

**DOI:** 10.3390/ph19040524

**Published:** 2026-03-24

**Authors:** Min Liu, Dongsheng Liu, Qian Zhang, Rui Zhang, Jiye Aa, Guangji Wang, Yuan Xie

**Affiliations:** 1 Key Laboratory of Drug Metabolism and Pharmacokinetics, State Key Laboratory of Natural Medicines, China Pharmaceutical University, Nanjing 210009, China; 3223071788@stu.cpu.edu.cn (M.L.); 3224071803@stu.cpu.edu.cn (D.L.); zq17613740396@163.com (Q.Z.); 3223071764@stu.cpu.edu.cn (R.Z.); jiyea@cpu.edu.cn (J.A.); 2 Research Unit of PK-PD Based Bioactive Components and Pharmacodynamic Target Discovery of Natural Medicine, Chinese Academy of Medical Sciences, Beijing 100700, China

**Keywords:** notoginsenoside R1, metabolic dysfunction-associated steatohepatitis, mitochondria, NDUFS2, OXPHOS

## Abstract

**Background:** Metabolic disorder-associated fatty liver disease (MASLD) is closely related to obesity and type 2 diabetes. Its pathogenesis involves many factors, including mitochondrial dysfunction, endoplasmic reticulum stress and intestinal flora disorders. Notoginsenoside R1 (NGR1) is a key bioactive component of *Panax notoginseng*. The purpose of this study was to investigate the therapeutic effect of notoginsenoside R1 (NGR1) on metabolic disorder-associated steatohepatitis (MASH) and its potential mechanism. **Methods:** Mice were fed a choline-deficient, L-amino acid-defined high-fat diet (CDAHFD) for 6 weeks and received NGR1 (50/100 mg/kg/day) in the last 3 weeks. The role of NGR1 was evaluated by developing metabolomics, proteomics and functional analysis. In addition, the effects of NGR1 on lipid droplet content, mitochondrial function and fatty acid oxidation in hepatocytes were also verified. **Results:** NGR1 improved MASH progression in CDAHFD-fed mice, significantly reduced liver triglyceride content from 31.2 ± 5.1 mmol/g to 20.5 ± 4.8 mg/g (*p* < 0.001), free fatty acid from 0.12 ± 0.03 mmol/g prot to 0.06 ± 0.028 mg/g (*p* < 0.001), TNF-α (*p* < 0.01), IL-1β (*p* < 0.001), α-SMA (*p* < 0.05) and Collagen1A1 levels (*p* < 0.01), as well as serum ALT and AST concentrations (*p* < 0.001), and alleviated hepatomegaly and lipid droplet accumulation. Metabolomics and proteomics analysis showed that NGR1 normalized liver metabolism in MASH mice and upregulated mitochondrial OXPHOS components, including NADH: ubiquinone oxidoreductase core subunit S2 (NDUFS2), and effectively reversed CDAHFD-induced mitochondrial dysfunction. Mitochondrial membrane potential and ATP production were restored. **Conclusions:** This study confirmed that NGR1 has significant therapeutic potential for MASH and improves mitochondrial function by upregulating NDUFS2. This study provides new insights for the future clinical treatment of MASH.

## 1. Introduction

Metabolic-dysfunction-associated steatotic liver disease (MASLD), strongly associated with obesity and type 2 diabetes, encompasses a spectrum from simple steatosis to steatohepatitis characterized by fat accumulation, inflammation and hepatocyte ballooning [1,2]. With a global prevalence of 32.4%, MASLD progression leads to fibrosis in severe cases, and 20% of MASH patients develop cirrhosis, liver failure or hepatocellular carcinoma within 20 years, making MASH a leading cause of liver transplantation [3,4,5]. Notably, 30–40% of liver-related deaths are associated with MASH progression [4,5]. MASH pathogenesis involves multiple factors including mitochondrial dysfunction, ER stress, inflammasome activation, and gut microbiota dysbiosis [6]. While lifestyle interventions can improve steatosis and fibrosis [7,8], their effects are often slow and limited, underscoring the need for pharmacotherapy—though current options like obeticholic acid remain inadequate [9]. This dilemma has changed with the approval of targeted drugs: in March 2024, the selective thyroid hormone receptor β agonist Resmetirom became the first drug approved by the FDA for the treatment of non-cirrhotic MASH adult patients with moderate to severe liver fibrosis (F2–F3) [10]. Based on the phase III MAESTRO-NASH test data, the 100 mg dose group achieved MASH remission in 29.9% of patients, and 25.9% of patients had liver fibrosis improvement ≥ 1 stage. And it can significantly reduce liver fat content and volume. At the same time, GLP-1 receptor agonist Semaglutide has not been directly approved for MASH indications, but its phase III ESSENCE clinical trial confirmed that after 72 weeks of treatment, liver inflammation can be reduced in 62.9% of patients and fibrosis does not deteriorate; fibrosis is also improved in 36.8% of patients, which provides an important choice for MASH treatment [11]. Even so, there is still room for improvement in existing treatments. For example, the improvement of fibrosis by remetinib is limited, and the efficacy of somaglutide in patients with cirrhosis is not yet clear. This significant unmet clinical need demands novel therapeutic strategies targeting MASH’s multifactorial nature, including hepatic injury, steatosis, inflammation and fibrosis.

Mitochondria, the cellular powerhouses, are central to energy metabolism through β-oxidation, TCA cycle and ATP production, while also regulating oxidative stress and innate immunity [12,13]. In MASH, mitochondrial dysfunction manifests as reduced mtDNA levels, impaired oxidative phosphorylation, and diminished oxygen consumption rates [14,15,16]. These abnormalities—including loss of membrane potential, depressed ATP synthesis and impaired biogenesis/fusion—directly contribute to MASH progression toward fibrosis and HCC [17], making mitochondrial restoration a promising therapeutic target. Hepatic fatty acids derive from adipose tissue lipolysis, intestinal chylomicron hydrolysis, and de novo lipogenesis [18]. These fatty acids are either oxidized in mitochondria, via CPT1/2-mediated transport and β-oxidation to acetyl-CoA, or esterified into triglycerides for VLDL secretion or lipid droplet storage [19,20]. Notably, while short/medium-chain fatty acids directly enter mitochondria, long-chain fatty acids require carnitine-dependent transport prior to β-oxidation [20].

Notoginsenoside, a bioactive compound derived from *Panax notoginseng*, has been utilized in Traditional Chinese Medicine (TCM) for over four centuries, primarily for its hemostatic and blood-activating properties according to classical pharmacopeias [21]. Modern pharmacological studies have identified its saponin components as key mediators of cardiovascular protection, demonstrating therapeutic potential against various circulatory disorders [22]. Clinically available formulations (e.g., Xuesaitong/Xueshuantong/Lulutong injections) have shown efficacy in attenuating myocardial injury, modulating inflammatory cascadesn, and inhibiting platelet aggregation [23]. Notoginsenoside R1 (NGR1), a key bioactive component of *Panax notoginseng*, demonstrates significant cardioprotective effects by reducing myocardial infarction size through TAK1-JNK/p38 inhibition and activating JAK2/STAT3 pathways to mitigate ischemia/reperfusion injury [24,25,26]. Additionally, NGR1 improves lipid metabolism via AKT/mTOR and AMPK activation while suppressing toxic lipid production [27,28]. In hepatic models, NGR1 exhibits hepatoprotective effects by inhibiting HSC activation through MAPK signaling, reducing inflammation via JNK pathway suppression, and ameliorating lipid metabolism disorders in ApoE-/- mice [29,30,31], suggesting therapeutic potential for MASH.

Although previous studies have confirmed that NGR1 has pharmacological activities such as anti-inflammatory and lipid-lowering, it has not yet revealed its therapeutic effect on MASH. This study aimed to clarify whether NGR1 improves mitochondrial function, thereby exerting a therapeutic effect on MASH. In this research, the anti-MASH effects of NGR1 were studied using a mouse model induced by a chronic CDAHFD. Based on this MASH model, NGR1’s hepatoprotective effects were studied, which include the regulation of hepatic lipid metabolism, reduction of inflammation, and inhibition of liver fibrosis. By employing metabolomics and proteomics techniques, we observed how NGR1 modulates the metabolic profile in MASH mice livers and revealed the molecular mechanisms underlying NGR1’s therapeutic effects on MASH.

## 2. Results

### 2.1. NGR1 Attenuates CDAHFD-Induced Hepatic Pathological Damage in Mice

**Figure 1:**
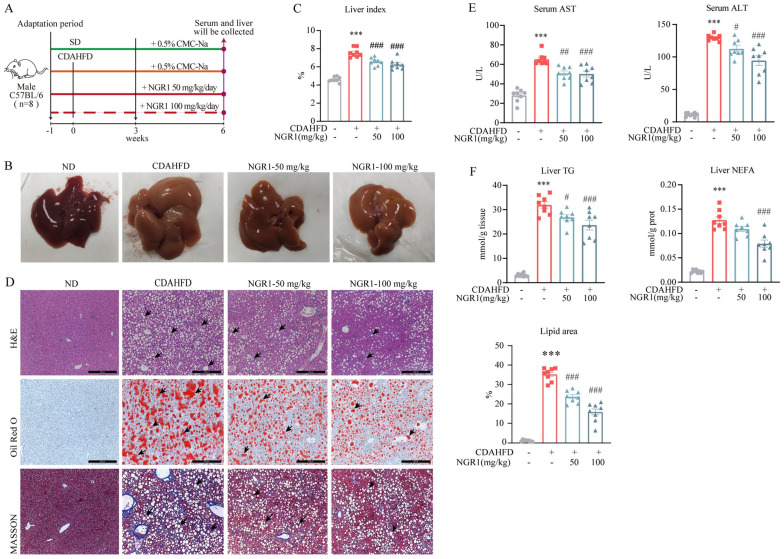
(**A**) Schematic representation of NGR1 intervention in CDAHFD−induced mice. (**B**) Liver picture (each group randomly selected one mouse liver to display). (**C**) Liver index (liver weight/body weight×100) (*n* = 8). (**D**) H&E staining, Oil Red O staining, and MASSON staining of mice in each group (each group randomly selected one mouse liver to display). Black arrows indicate hepatic lipid vacuoles and lipid droplets. The proportion of lipid area in each group is shown (the area was calculated using Imagine J 1.53t software) (*n* = 8). The stained sections were observed by Leica DMI3000 B research microscope at 200 magnifications (*n* = 8). (**E**) Serum AST and Serum ALT (*n* = 8). (**F**) Liver TG and Liver NEFA (*n* = 8). Data are expressed as the mean ± SE. *** *p* < 0.001 vs. the control group, # *p* < 0.05, ## *p* < 0.01, ### *p* < 0.001 vs. the model group.

A MASH mouse model was established by feeding the animals a CDAHFD for 6 weeks, followed by intragastric administration of NGR1 (50 mg/kg/day or 100 mg/kg/day) during the last 3 weeks (Figure 1A). Macroscopic liver examination revealed that the CDAHFD induced hepatomegaly, with livers exhibiting a yellowish-brown, rough, and granular surface. NGR1 treatment markedly ameliorated these morphological abnormalities (Figure 1B). The liver index was significantly elevated in the CDAHFD group but decreased after NGR1 administration, confirming its ability to mitigate diet-induced hepatomegaly (Figure 1C). In addition, compared with the control group, model mice showed significant weight loss and reduced food intake. NGR1 treatment significantly alleviated CDAHFD-induced weight loss and decreased food intake, suggesting that NGR1 improves CDAHFD-induced metabolic homeostasis disruption in mice. (Figure S1). These results suggest that NGR1 alleviates CDAHFD-induced liver morphological damage and volume expansion.

Histopathological analysis of H&E-stained liver sections demonstrated that the model group exhibited increased hepatic lipid vacuolization, severe lipid accumulation, and inflammatory cell infiltration compared to controls. In contrast, NGR1 treatment (50 and 100 mg/kg) reduced lipid vacuoles, attenuated hepatic steatosis, and suppressed inflammatory cell infiltration. Oil Red O staining further confirmed that the CDAHFD significantly increased hepatic lipid deposition, while NGR1 administration dose-dependently reduced triglyceride accumulation. Additionally, MASSON staining revealed elevated collagen fiber deposition and aggravated fibrosis in the model group, both of which were ameliorated by NGR1 treatment (Figure 1D). Collectively, these findings indicate that the CDAHFD successfully induced MASH in mice, characterized by hepatic steatosis, inflammation, and fibrosis, and that NGR1 effectively counteracted these pathological changes.

To further assess NGR1’s pharmacodynamic effects, serum and hepatic biochemical markers were analyzed. Liver injury triggers the release of AST and ALT into the bloodstream, while NGR1 significantly suppressed the CDAHFD-induced elevation of serum transaminases (Figure 1E). Consistent with histopathological observations, NGR1 treatment reduced hepatic triglyceride (TG) and non-esterified fatty acid (NEFA) levels, with a more pronounced lipid-lowering effect at the 100 mg/kg dose (Figure 1F). These results demonstrate that NGR1 not only mitigates liver injury but also improves hepatic lipid metabolism.

### 2.2. NGR1 Reduces Liver Lipid Synthesis, Inflammation, and Fibrosis in CDAHFD-Fed Mice

**Figure 2:**
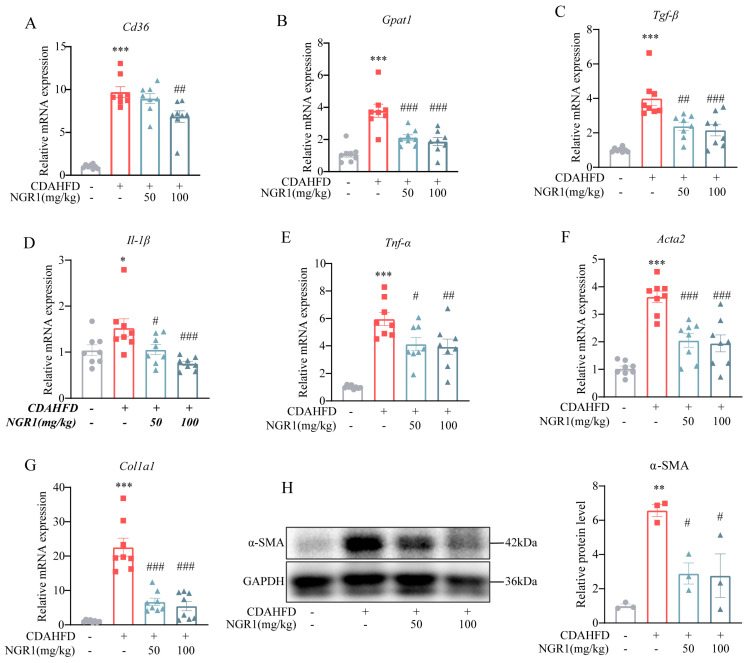
Relative mRNA expression of (**A**) Cd36, (**B**) Gpat, (**C**) Tgf−β, (**D**) Il−1β, (**E**) Tnf−α, (**F**) Acta2 and (**G**) Clo1a1 in the liver (*n* = 8). (**H**) Immunoblotting analysis of α-SMA in the liver, and bar graph displays quantification of blots of α-SMA (*n* = 3). Data are expressed as the mean ± SEM. * *p* < 0.05, ** *p* < 0.01, *** *p* < 0.001 vs. the control group, # *p* < 0.05, ## *p* < 0.01, ### *p* < 0.001 vs. the model group.

In MASH pathogenesis, upregulated expression of fatty acid translocase CD36 and glycerol-3-phosphate acyltransferase 1 (GPAT1), a key enzyme in triglyceride synthesis, promotes hepatic lipid accumulation [32,33]. NGR1 administration significantly downregulated mRNA levels of both *Cd36* and *Gpat1*, suggesting its regulatory role in lipid synthesis-related gene expression (Figure 2A,B).

To evaluate NGR1’s effect on hepatic inflammation in MASH mice, we analyzed inflammatory gene expression using quantitative reverse transcription PCR (qRT-PCR). The CDAHFD group showed markedly elevated expression of pro-inflammatory genes, including *Tgf-β*, *Il-1β*, and *Tnf-α*, which was significantly attenuated by NGR1 treatment (Figure 2C–E). Since hepatic inflammation promotes fibrogenesis, we further examined fibrosis-related gene expression. qRT-PCR revealed that CDAHFD feeding upregulated the expression of *Acta2* and *Col1a1*, while NGR1 administration effectively suppressed their expression (Figure 2F,G). Western blot analysis confirmed that NGR1 reduced α-smooth muscle actin (α-SMA) protein levels, demonstrating its anti-fibrotic effects (Figure 2H). Collectively, these results indicate that NGR1 ameliorates MASH by modulating key inflammatory and fibrotic mediators at both transcriptional and translational levels.

### 2.3. Metabolic Profile Characterization in Liver and Serum of MASH Mice

**Figure 3:**
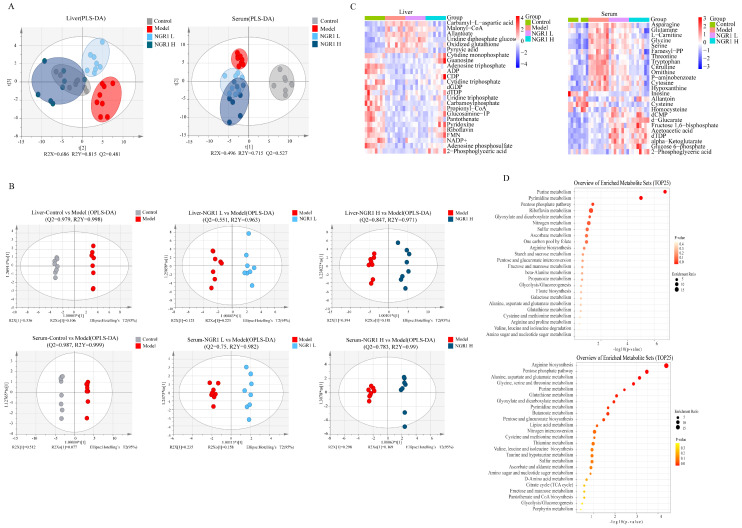
(**A**) Partial least squares discrimination analysis (PLS−DA) of metabolites in each group of mice. PLS−DA of the liver and PLS−DA of the serum. (**B**) Orthogonal projections to latent structures discriminant analysis (OPLS−DA) score plot of metabolites in each group of mice. (**C**) Heatmap of metabolites in each group of mice. NGR1 L represented the NGR1−50 mg/kg group, NGR1 H represented the NGR1−100 mg/kg group. (**D**) KEGG pathway enrichment analysis of metabolites in each group of mice.

Partial least squares-discriminant analysis (PLS-DA) of hepatic and serum metabolites revealed distinct metabolic patterns among experimental groups (Figure 3A). The model group showed significant metabolic deviation from controls, demonstrating CDAHFD-induced metabolic dysregulation. NGR1 treatment dose-dependently shifted these metabolic profiles toward the control state, with more pronounced correction observed in the high-dose group, indicating NGR1’s capacity to ameliorate CDAHFD-induced metabolic disturbances. Further validation through orthogonal projections to latent structures-discriminant analysis (OPLS-DA) revealed distinct metabolic clustering patterns across all experimental groups (Figure 3B). The control and model groups showed clear separation in both hepatic and serum metabolic profiles, while NGR1-treated groups formed intermediate clusters between these extremes, demonstrating substantial metabolic reprogramming following intervention.

Differential metabolite screening (RSD < 0.3, FC > 1.2 or < 0.84, *p* < 0.05) and subsequent heatmap analysis of the top 25 significantly altered metabolites revealed NGR1’s multifaceted metabolic effects (Figure 3C). In liver tissue, NGR1 administration counteracted CDAHFD-induced elevations in glycolipid metabolites while restoring depleted nucleotide pools. Serum analysis showed NGR1 effectively normalized dysregulated amino acid metabolism, reducing elevated levels of asparagine, glutamine, glycine, and serine while rescuing depressed concentrations of cysteine and homocysteine. KEGG pathway enrichment analysis of these differential metabolites identified several key metabolic pathways modulated by NGR1 (Figure 3D). In liver tissue, notoginsenoside R1 administration reduced the upregulation of glucose and lipid metabolism-related metabolites such as malonyl CoA, uridine diphosphate glucose and pyruvate induced by CDAHFD and increased the downregulation of purine and pyrimidine nucleotide metabolites such as ATP, ADP, CDP and CTP induced by CDAHFD, suggesting that notoginsenoside R1 is directly involved in improving the abnormality of liver purine/pyrimidine metabolic pathway by reversing the disorder of these metabolites and improving the disorder of liver glucose and lipid metabolism induced by CDAHFD. Serum analysis showed that notoginsenoside R1 administration significantly reduced the upregulation of amino acid metabolites such as asparagine, glutamine, glycine, and serine, and increased the downregulation of amino acid metabolites such as cysteine and homocysteine, suggesting that notoginsenoside R1 regulates these amino acids and directly participates in the repair of arginine biosynthesis and glutathione metabolic pathways, thereby improving serum amino acid metabolic disorders. Hepatic effects involved regulation of purine/pyrimidine metabolism, pentose phosphate pathway, riboflavin metabolism, and folate-mediated one-carbon metabolism. Systemic effects included modulation of arginine biosynthesis, glutathione metabolism, and additional involvement in pentose phosphate and nucleotide metabolic pathways. These comprehensive metabolic improvements suggest NGR1 exerts its therapeutic effects against MASH through multi-target regulation of interconnected metabolic networks in both hepatic and systemic compartments.

### 2.4. Proteomic Analysis of Livers of CDAFHD-Fed Mice

**Figure 4:**
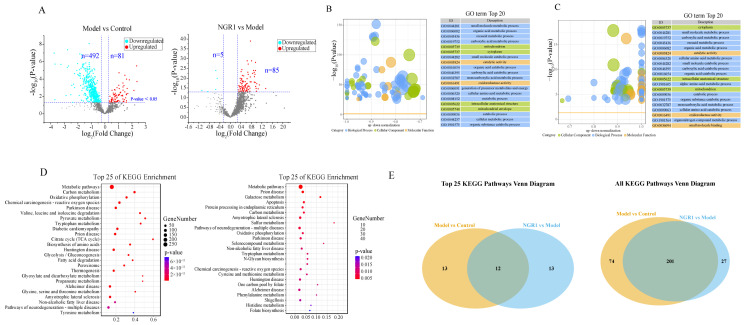
(**A**) Volcano plot that shows differentially expressed proteins with statistical significance and fold change between different groups. Significant proteins were selected by FC > 1.2 or < 0.84 and *p* < 0.05. (**B**) Bubble plot of significantly enriched GO terms in the model vs. control comparative datasets. The cut−off for labeling was set at an adjusted *p*−value of 0.05. The horizontal axis indicates the given terms are more likely to be downregulated. The area of circles is proportional to the number of proteins assigned to the term. (**C**) Bubble plot of significantly enriched GO terms in the NGR1 vs. model comparative datasets. The cut−off for labeling was set at an adjusted *p*−value of 0.05. The horizontal axis indicates the given terms are more likely to be upregulated. The area of circles is proportional to the number of proteins assigned to the term. (**D**) KEGG enrichment analysis between different groups. The cut−off for labeling was set at an adjusted *p*−value of 0.05. The horizontal axis indicates the given terms are more likely to be upregulated. The area of circles is proportional to the number of proteins assigned to the term. (**E**) KEGG pathway Venn diagram analysis between different groups: The left panel (Top 25 KEGG Pathways Venn diagram) shows the overlapping and unique top 25 KEGG pathways of differential metabolite enrichment in ‘model vs. control’ and ‘NGR1 vs. model’; the right panel (All KEGG Pathways Venn diagrams) shows all the enriched KEGG pathways.

Comprehensive proteomic profiling of liver samples from control, CDAHFD model, and high-dose NGR1 (100 mg/kg) treatment groups was performed using proteomics technology. Protein identification was conducted against the Uniprot mouse protein database, with subsequent bioinformatics analysis to elucidate NGR1’s potential regulatory pathways and the functional implications of differentially expressed proteins. Applying stringent criteria (FC > 1.2 or < 0.84, *p* < 0.05), we identified significant proteomic alterations between groups (Figure 4A). Compared to controls, CDAHFD feeding induced profound changes, with 81 proteins significantly upregulated and 492 downregulated. Notably, NGR1 treatment reversed this pattern.

Gene Ontology (GO) analysis of model versus control groups revealed widespread functional dysregulation across all categories (Figure 4B,C). Biological processes (BP) showed marked downregulation of metabolic pathways including organic acid and carboxylic acid metabolism, cellular amino acid metabolism, and energy production pathways. Cellular component (CC) analysis demonstrated significant depletion of mitochondrial proteins and cytoplasmic constituents, while molecular function (MF) categories exhibited reduced catalytic and oxidoreductase activities. These findings collectively indicate that NGR1 ameliorates CDAHFD-induced MASH through comprehensive modulation of hepatic metabolic networks. KEGG pathway enrichment analysis provided further mechanistic insights (Figure 4D). At the metabolic pathway level, there was a significant pathway association between the model vs. control group and the NGR1 vs. model group: from the top 25 pathway, a total of 12 overlapping pathways were enriched in the two groups, suggesting that NGR1 administration can target the core pathway of abnormal changes in the model group. From the perspective of all pathways, 201 pathways were the core pathways involved in the two groups compared with the control group (74 pathways), and the administration group compared with the model group (27 pathways), which further indicated that NGR1 could reverse the abnormal metabolic pathways of MASH model mice, also providing pathway-level support for its mechanism of improving MASH (Figure 4E). The model group showed significant enrichment in one-carbon metabolism, oxidative phosphorylation, pyruvate metabolism, TCA cycle, and fatty acid degradation pathways. Following NGR1 treatment, pathway analysis revealed distinct enrichment patterns in galactose metabolism, one-carbon metabolism, and oxidative phosphorylation, suggesting these as potential therapeutic targets for NGR1’s anti-MASH effects.

### 2.5. Analysis of Mitochondrial Function-Related Differential Metabolites and Proteins

**Figure 5:**
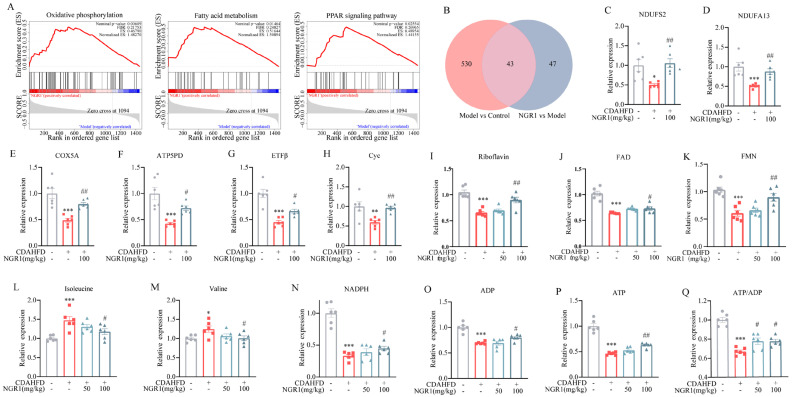
(**A**) Gene Set Enrichment Analysis (GSEA) enrichment analysis between NGR1 group and model group. GSEA showed that NGR1 administration is positively correlated with oxidative phosphorylation, fatty acid metabolism, and PPAR signaling pathway. (**B**) Venn plot showing the common proteins in the liver of three groups. Relative expression of (**C**) NDUFS2, (**D**) NDUFA13, (**E**) COX5A, (**F**) ATP5PD, (**G**) ETFβ and (**H**) CYC in the liver. Data are expressed as the mean ± SEM, *n* = 6 in each group. * *p* < 0.05, ** *p* < 0.01, *** *p* < 0.001 vs. the control group, # *p* < 0.05, ## *p* < 0.01 vs. the model group. Relative expression of (**I**) riboflavin, (**J**) FAD and (**K**) FMN in the liver. Data are expressed as the mean ± SEM, *n* = 6 in each group. *** *p* < 0.001 vs. the control group, # *p* < 0.05, ## *p* < 0.01 vs. the model group. Relative expression of (**L**) Isoleucine and (**M**) Valine in the liver. Data are expressed as the mean ± SEM, *n* = 6 in each group. * *p* < 0.05, *** *p* < 0.001 vs. the control group, # *p* < 0.05 vs. the model group. Relative expression of (**N**) NADPH, (**O**) ADP and (**P**) ATP in the liver. (**Q**) The ratio of ATP/ADP in the liver. Data are expressed as the mean ± SEM, *n* = 6 in each group. *** *p* < 0.001 vs. the control group, # *p* < 0.05, ## *p* < 0.01 vs. the model group.

Gene Set Enrichment Analysis (GSEA) of hepatic proteins revealed significant enrichment of oxidative phosphorylation, fatty acid metabolism, and PPAR signaling pathways following NGR1 treatment (Figure 5A), suggesting these pathways as key mediators of NGR1’s therapeutic effects. Both KEGG and GSEA analyses consistently identified oxidative phosphorylation as the most significantly enriched pathway, indicating that NGR1 ameliorates MASH through modulation of mitochondrial function. This finding aligns with the established literature demonstrating the critical involvement of mitochondrial function in fatty acid metabolism and PPAR signaling pathways [34]. The observed enrichment of fatty acid metabolism and PPAR signaling pathways in our GSEA results supports the hypothesis that NGR1 regulates hepatic lipid metabolism through mitochondrial-mediated mechanisms. Venn diagram analysis identified 43 overlapping differentially expressed proteins between the model–control and treatment–model comparisons (Figure 5B), representing potential key mediators of NGR1’s therapeutic effects. Notably, NGR1 treatment reversed CDAHFD-induced downregulation of mitochondrial complex I subunits, including NDUFS2 (Figure 5C) and NDUFA13 (Figure 5D), and restored expression of mitochondrial complex IV COX5A (Figure 5E) and complex V ATP5PD (Figure 5F). Furthermore, NGR1 significantly upregulated key electron transport chain components ETFβ (Figure 5G) and CYC (Figure 5H) that were suppressed by CDAHFD, thereby enhancing mitochondrial electron transfer efficiency. These findings collectively demonstrate that NGR1 improves mitochondrial function through coordinated upregulation of respiratory chain complexes and electron transport proteins.

KEGG analysis revealed NGR1’s significant impact on hepatic riboflavin metabolism. Given that riboflavin deficiency impairs mitochondrial electron transport through FAD depletion [35], the NGR1-mediated increase in hepatic FAD content (Figure 5I) represents a crucial mechanism for restoring mitochondrial function. Importantly, mitochondrial complex I—which contains FMN and iron–sulfur clusters critical for its activity [36]—showed significantly improved function following NGR1 treatment, corresponding with elevated hepatic FMN levels (Figure 5J). These results indicate that NGR1 enhances mitochondrial electron transport through modulation of flavin metabolism. In addition, NGR1 treatment significantly reduced hepatic isoleucine and valine levels (Figure 5L,M), suggesting restoration of β-oxidation capacity. Additionally, while CDAHFD caused marked depletion of hepatic NADPH (Figure 5N), NGR1 effectively restored NADPH levels, thereby mitigating oxidative stress and inflammation. Finally, NGR1 reversed CDAHFD-induced depletion of adenine nucleotides, significantly increasing both ADP and ATP levels while improving the ATP/ADP ratio (Figure 5Q), indicating enhanced mitochondrial ATP production capacity.

### 2.6. NGR1 Ameliorates Hepatocyte Steatosis and Mitochondrial Dysfunction in MASH Mice

**Figure 6:**
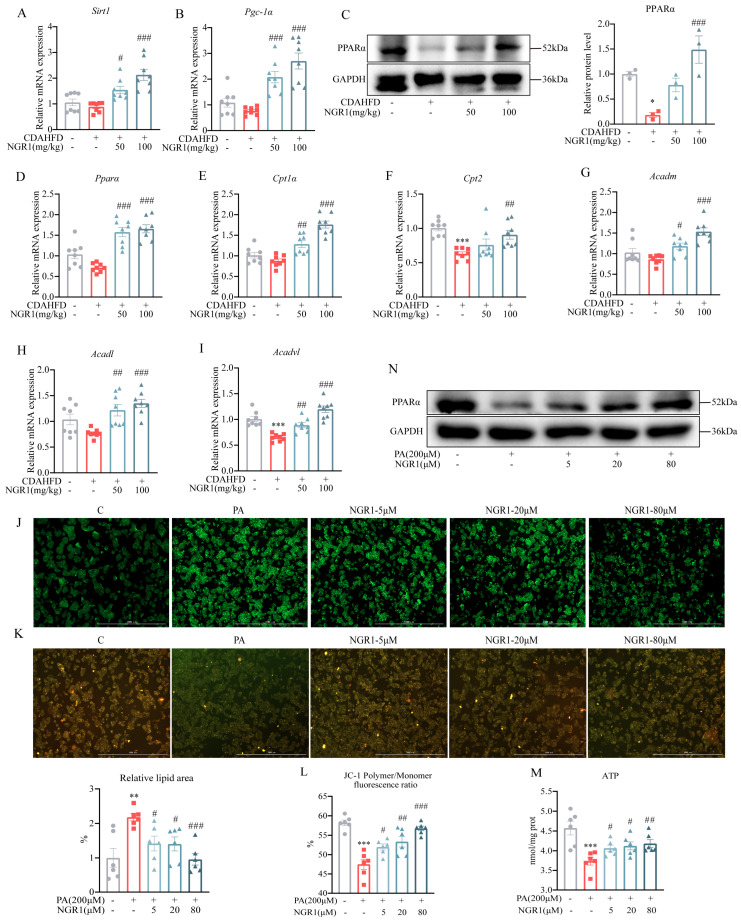
Relative mRNA expression of (**A**) Sirt1 and (**B**) Pgc−1α in the liver. Data are expressed as the mean ± SEM, *n* = 8 in each group. # *p* < 0.05, ### *p* < 0.001 vs. the model group. (**C**) Immunoblotting analysis of PPARαin the liver, and the bar graph displays quantification of blots of PPARα (*n* = 3). Relative mRNA expression of (**D**) Pparα, (**E**) Cpt1α, (**F**) Cpt2, (**G**) Acadm, (**H**) Acadl and (**I**) Acadvl in the liver. Data are expressed as the mean ± SEM, *n* = 8 in each group. * *p* < 0.05, *** *p* < 0.001 vs. the control group, # *p* < 0.05, ## *p* < 0.01, ### *p* < 0.001 vs. the model group. (**J**) Representative photomicrographs of BODIPY staining at 4× magnification and quantitative results of BODIPY staining. Data are expressed as the mean ± SEM, *n* = 6 in each group. ** *p* < 0.01 vs. the control group, # *p* < 0.05, ### *p* < 0.001 vs. the model group. (**K**) Photograph showing JC−1 red and JC−1 green merged image at 4× magnification. (**L**) Numerical data express the proportion of red/green (Polymer/Monomer) fluorescence. (**M**) The changes in intracellular ATP levels. Data are expressed as the mean ± SEM, *n* = 6 in each group. *** *p* < 0.001 vs. the control group, # *p* < 0.05, ## *p* < 0.01, ### *p* < 0.001 vs. the model group. (**N**) Immunoblotting analysis of PPARα in the PA−induced HepG2 cells.

SIRT1, a class III histone deacetylase, plays a pivotal role in mitochondrial homeostasis. SIRT1-mediated deacetylation of PGC-1α—the central coordinator of mitochondrial biogenesis and metabolism—activates transcriptional programs governing mitochondrial function and fatty acid oxidation, thereby maintaining cellular bioenergetics [37]. Our qRT-PCR analysis revealed that NGR1 treatment significantly upregulated hepatic *Sirt1* and *Pgc-1α* mRNA expression (Figure 6A,B), demonstrating its capacity to enhance mitochondrial quality control and biogenesis pathways compromised by CDAHFD. PPARα, the nuclear receptor governing mitochondrial fatty acid β-oxidation, exerts its effects through transcriptional activation of rate-limiting enzymes CPT1α and CPT2 [38]. In MASH mice, NGR1 treatment robustly elevated hepatic PPARα protein expression and upregulated the expression of its downstream targets *Cpt1α* and *Cpt2*, thereby restoring impaired fatty acid β-oxidation capacity. The mitochondrial β-oxidation cascade initiates with acyl-CoA dehydrogenase-mediated reactions. We observed that NGR1 significantly increased mRNA levels of key dehydrogenases, including *Acadm* for medium-chain, *Acadl* for long-chain, and *Acadvl* for very-long-chain (Figure 6G–I). This multi-target upregulation enhances the initial, rate-limiting steps of fatty acid catabolism, facilitating complete β-oxidation to acetyl-CoA.

In palmitate (PA)-treated HepG2 cells, we quantified lipid accumulation via BODIPY™ 493/503 staining. PA treatment (200 μM, 24 h) markedly increased neutral lipid deposition (Figure 6J), which was dose-dependently attenuated by NGR1. Mitochondrial dysfunction, a hallmark of hepatic lipotoxicity, was assessed using JC-1 potentiometric dye. NGR1 treatment restored the PA-induced collapse of mitochondrial membrane potential, as evidenced by JC-1 aggregate/monomer ratio (Figure 6K,L). Concordantly, NGR1 reversed PA-induced ATP depletion (Figure 6M), confirming functional recovery of oxidative phosphorylation. Western blot analysis revealed that NGR1 counteracted PA-induced downregulation of PPARα protein (Figure 6N), mechanistically explaining its pro-β-oxidation effects.

### 2.7. NGR1 Improves Mitochondrial Homeostasis

**Figure 7:**
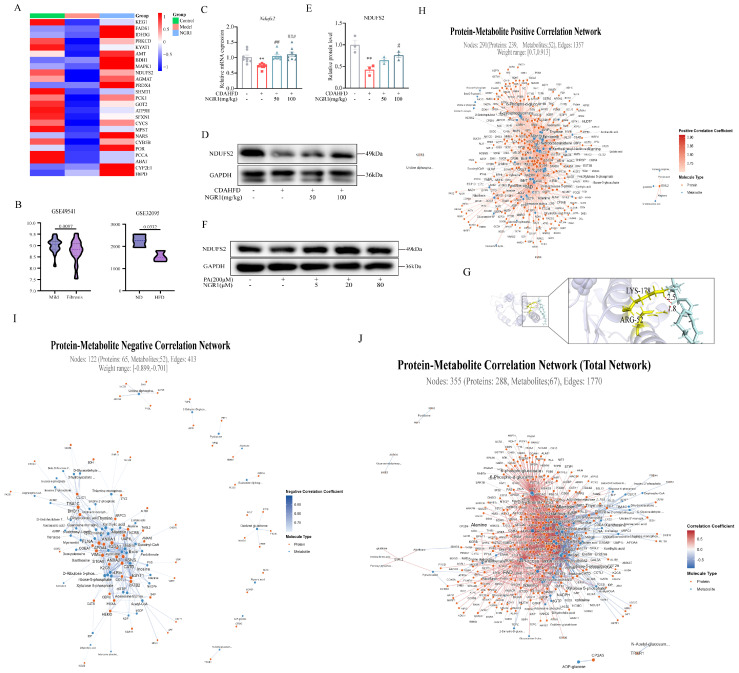
(**A**) Heatmap of average mitochondria−associated proteins in the liver of three groups. (**B**) The expression of NDUFS2 in publicly available Gene Expression Omnibus (GEO) datasets was analyzed. (**C**) Relative mRNA expression of Ndufs2 in the liver (*n* = 8). (**D**) Immunoblotting analysis of NDUFS2 in the liver. (**E**) Bar graph displays quantification of blots of NDUFS2 (*n* = 3). Data are expressed as the mean ± SEM. ** *p* < 0.01 vs. the control group, # *p* < 0.05, ## *p* < 0.01, ### *p* < 0.001 vs. the model group. (**F**) Immunoblotting analysis of NDUFS2 in the PA−induced HepG2 cells. (**G**) Molecular docking diagram of NGR1 and NDUFS2. (**H**) Interconnectivity of differentially expressed proteins and metabolites in the positive correlation network. (**I**) Interconnectivity of differentially expressed proteins and metabolites in the negative correlation network. (**J**) Interconnectivity of differentially expressed proteins and metabolites in the correlation network. The screening conditions were as follows: positive correlation r ≥ 0.7, negative correlation r ≤ −0.7, FDR < 0.05. Data are expressed as the mean ± SEM. ** *p* < 0.01 vs. the control group, # *p* < 0.05, ## *p* < 0.01, ### *p* < 0.001 vs. the model group.

Our findings demonstrate that NGR1 effectively mitigates mitochondrial dysfunction, enhances fatty acid β-oxidation, and reduces lipid accumulation in both in vivo and in vitro MASH models. To elucidate the underlying molecular mechanisms, we performed heatmap analysis of the top 25 differentially expressed mitochondrial proteins between NGR1-treated and model groups (Figure 7A). NDUFS2, a core subunit of mitochondrial complex I, showed significant downregulation in the model group, which was reversed by NGR1 treatment. Examination of GEO datasets (GSE49541) showed progressive reduction in hepatic NDUFS2 expression from steatosis to MASH in human patients. This pattern was recapitulated in murine models, and high-fat diet (GSE32095) treatments led to significant NDUFS2 suppression (Figure 7B). Our experimental data confirmed these observations, with qRT-PCR and Western blot analyses demonstrating that NGR1 effectively restored NDUFS2 expression at both transcriptional and translational levels in liver tissues from MASH mice (Figure 7C–E). Parallel results were obtained in cellular models, where PA-induced NDUFS2 downregulation in HepG2 cells was consistently reversed by NGR1 treatment (Figure 7F). This observation indicates that NDUFS2 deficiency in MASH impairs mitochondrial complex assembly, reduces ATP production, and contributes to overall mitochondrial dysfunction, suggesting its pivotal role in MASH pathogenesis.

The binding mode predicted by molecular docking tools showed that NGR1 could form a stable interaction with NDUFS2; among them, there are hydrogen bonds between NGR1 and specific amino acid residues of NDUFS2 (such as Lys-178 and Arg-52), as marked by red dotted lines. These hydrogen bonds represent one of the key forces maintaining the binding stability between NGR1 and NDUFS2 (Figure 7G). We further carried out the correlation analysis between proteome and metabolome. The results showed that the positive synergistic regulation relationship between proteins and metabolites exhibited sparsity and specificity. A subset of molecules had strong positive correlation, which reflected the precise targeting of molecular regulation in this biological process (Figure 7H). In contrast, negative antagonistic relationships between the two shows stronger sparsity and specificity (Figure 7I). From the perspective of overall network characteristics, positive correlation edges accounted for 76.7% (1357 in total) and negative correlation edges accounted for 23.3% (413 in total), indicating that the interaction between proteins and metabolites in this biological process was centered on positive synergistic regulation and assisted by negative antagonistic regulation (Figure 7J). Meanwhile, the results of network density (0.092) and average connectivity (10.0) further verified that the significant molecular correlations are not widespread but are concentrated in specific molecular combinations, reflecting the high specificity of the protein–metabolite regulatory network in this process.

## 3. Discussion

The choline-deficient, L-amino acid-defined high-fat diet (CDAHFD) has become an effective way to construct a stable and reliable MASH animal model [39,40]. While conventional high-fat diets primarily induce simple steatosis and metabolic disturbances [39], CDAHFD uniquely combines methionine restriction (0.1%) with complete choline deficiency to accelerate MASH progression through impaired VLDL secretion and subsequent hepatic triglyceride accumulation, while avoiding the severe weight loss associated with complete methionine-choline deficient diets [41,42]. Our findings confirm and extend these previous observations by demonstrating that six weeks of CDAHFD feeding in C57BL/6J mice reliably produces the full spectrum of MASH pathology, including steatosis, inflammation, and fibrosis. These findings are consistent with previous reports and further validate the reliability of the CDAHFD-induced MASH model.

Previous research has identified several key molecular mechanisms underlying MASH pathogenesis, including CD36-mediated fatty acid uptake [43,44], GPAT1-driven de novo lipogenesis [45], and cytokine-induced inflammation and fibrosis [46,47]. Through integrated metabolomic and proteomic analyses, we have identified previously unrecognized pathways through which NGR1 exerts its therapeutic effects. These findings move beyond a single-target perspective and reveal a multi-faceted regulatory network. By simultaneously modulating key nodes involved in lipid metabolism, mitochondrial function, inflammatory responses, and fibrogenesis, our work provides a more complete understanding of its mechanism of action. Collectively, these results position NGR1 as a promising multi-target therapeutic candidate capable of addressing the complex metabolic, inflammatory, and fibrotic aspects of MASH pathogenesis, underscoring its potential to correct the underlying metabolic dysregulation that drives disease progression.

Studies have shown that the occurrence and development of MASLD/MASH are closely related to nucleotide metabolism, amino acid homeostasis and mitochondrial dysfunction [46,47,48], including purine/pyrimidine metabolic disorders and elevated branched-chain amino acids (BCAAs) [46,47], which are highly similar to the metabolic characteristics of human patients. In this study, non-targeted metabolomics analysis confirmed that CDAHFD can significantly disrupt the liver and serum metabolic profiles, and NGR1 can systematically reverse above-abnormal changes. Firstly, NGR1 restores the biosynthesis of nucleotides by synergistically regulating the pentose phosphate pathway and folic acid-mediated one-carbon metabolism, and its regulatory range is more comprehensive than the previously reported single-target intervention [48]. Secondly, NGR1 significantly reduces the accumulation of BCAAs, and its effect is better than that of conventional intervention [49]. This process may be related to the enhancement of acyl-CoA dehydrogenase activity. Thirdly, NGR1 increases the level of riboflavin-related cofactors and promotes ATP production, significantly improving mitochondrial bioenergy metabolism disorders. The above results show that NGR1 can correct nucleotide defects, amino acid imbalance and mitochondrial dysfunction at the same time. It is a natural small molecule with multi-target and multi-pathway regulation advantages, which provides a new strategy for solving the complex metabolic network disorder of MASH.

Liver mitochondria are the core organelles that regulate fatty acid oxidation, tricarboxylic acid cycle and ATP synthesis, and their dysfunction is a key driver of MASH. Oxidative phosphorylation (OXPHOS) is the core pathway of mitochondrial energy generation, and its decreased activity directly leads to abnormal lipid metabolism and enhanced oxidative stress [50]. In this study, proteomics, KEGG and GSEA confirmed that NGR1 could significantly upregulate the protein level of OXPHOS-related pathways and increase the content of mitochondrial metabolites, suggesting that NGR1 could enhance mitochondrial respiratory efficiency and improve energy production. The mitochondrial protective properties of NGR1 have been well-documented in other cell types, including neurons and osteoblasts, where it has been shown to reduce mitochondrial damage, restore ATP levels, stabilize membrane potential, and mitigate oxidative stress [51,52]. The results of the present study align closely with these established effects. Importantly, we expand upon these prior observations by providing the first evidence that NGR1 exerts similar protective actions on hepatic mitochondria, thereby ameliorating the energy metabolism dysfunction central to MASH pathogenesis.

At the same time, GSEA results showed that NGR1 significantly enriched fatty acid metabolism and the PPAR signaling pathway. PPARα is a key nuclear receptor for fatty acid oxidation in liver mitochondria, and its loss of function can directly lead to hepatic steatosis [53]. This study suggests that NGR1 may promote the oxidative decomposition of fatty acids by activating the PPARα pathway, thereby reducing excessive lipid accumulation, which is highly consistent with the classical metabolic regulation function of PPARα.

NDUFS2 is the core subunit of mitochondrial respiratory chain complex I, which is essential for maintaining the assembly, stability and catalytic activity of complex I. Previous studies have shown that NDUFS2 knockdown can significantly inhibit ATP synthesis, reduce mitochondrial membrane potential and promote oxidative stress [54]. In this study, molecular docking analysis confirmed that NGR1 can form a stable binding conformation with NDUFS2, suggesting that there is a potential direct interaction between the two; at the same time, combined with the combined correlation analysis of proteomics and metabolomics, it was further verified that NGR1 could affect mitochondrial energy metabolism and lipid metabolism-related pathways by regulating NDUFS2. More importantly, this study confirmed for the first time that NGR1 can restore NDUFS2 expression, thereby improving mitochondrial membrane potential, increasing ATP production, reducing ROS accumulation, and reversing fatty acid oxidation disorders. The above results indicate that NDUFS2 is a key target for NGR1 to exert mitochondrial protection and anti-MASH effects, providing a new potential direction for targeted intervention of MASH.

In this study, we systematically elucidated the molecular mechanism by which NGR1 ameliorates MASH through an integrated approach combining multi-omics analysis with cellular functional validation. For the first time, we reveal a novel NGR1–NDUFS2–mitochondrial regulatory axis. The use of the CDAHFD mouse model, which closely recapitulates human MASH pathology, along with a comprehensive evidence chain, underscores the mechanistic innovation and highlights the strong fundamental research value and translational potential of this work.

Nevertheless, several limitations should be acknowledged. This study is primarily based on animal and cellular experiments, and the upstream molecular mechanisms through which NGR1 regulates NDUFS2 have not been fully elucidated. Moreover, systematic pharmacokinetic profiling and long-term safety evaluation have yet to be conducted, and further translational studies are required before clinical application can be considered. Importantly, however, the key pathological alterations observed in this study, including metabolic dysregulation, mitochondrial dysfunction, and aberrant NDUFS2 expression, are highly consistent with features of human MASH. These findings suggest that NGR1 holds promise as a natural mitochondrial-targeting candidate for MASH therapy.

Overall, our results provide an important theoretical foundation and valuable reference for future research into the pathogenesis of human MASH, as well as for target identification and the development of clinical intervention strategies.

## 4. Materials and Methods

### 4.1. Animal Experiment Protocol

Male SPF-grade C57BL/6JNifdc mice were purchased from Zhejiang Viton Lihua Experimental Animal Technology Co., Ltd. (Zhejiang, China). The mice were housed in standard cages and a 12 h/12 h light/dark cycle. After a 1-week acclimatization period with free access to food and water, 32 mice were randomly divided into four groups (*n* = 8). The control group was fed with a standard diet for 6 weeks, followed by daily gavage of 0.5% CMC-Na solution (0.1 mL/10 g body weight) for the final 3 weeks. The model group was fed with a choline-deficient, L-amino acid-defined high-fat diet (CDAHFD) for 6 weeks, with daily 0.5% CMC-Na gavage (0.1 mL/10 g) during the last 3 weeks. The NGR1 groups (50 or 100 mg/kg, equivalent to human dose of 11.71 or 23.42 mg/kg/day) were fed with CDAHFD for 6 weeks, followed by daily gavage of 5 or 10 mg/mL NGR1 suspension (0.1 mL/10 g) for the final 3 weeks. The CDAHFD contained 60% calories from fat, 0.1% methionine (incomplete deficiency), and no added choline.

The body weight of the mice was measured twice a week and recorded. The animals were fasted for 12 h the day before sacrifice. After intraperitoneal injection of pentobarbital sodium (2.5 mg/100 g BW), the mice were anesthetized, and the blood of the mice was collected. The serum was separated and stored in a −80 °C low-temperature refrigerator for subsequent testing. After the mice were sacrificed, the livers of the mice were collected, weighed and photographed. Part of the liver tissue was fixed in 4% paraformaldehyde fixative for the preparation of paraffin sections. Some liver tissues were embedded with OCT for the preparation of frozen sections. The remaining liver tissue was stored in a refrigerator at −80 °C for subsequent detection.

### 4.2. Cell Culture

HepG2 cells were cultured in a 5% CO_2_ incubator supplemented with 10% fetal bovine serum (FBS, 10099, Gibco, Miami, FL, USA) and 1% penicillin-streptomycin (15140122, Gibco). HepG2 cells were treated with medium containing 0.2 mM palmitic acid (PA, P0500, Sigma Aldrich, St. Louis, MO, USA) for the times indicated, and fatty acid-free bovine serum albumin was used as a control. After the HepG2 cells were plated, the drug was administered when the cell density reached 70–80%. The blank solvent (10% BSA solution) was added to the control hole, the palmitic acid solution was added to the model group, and the palmitic acid solution was added to the administration group. At the same time, 5 μM and 20 μM were given.

### 4.3. Histological Analysis

The paraffin-embedded paraformaldehyde-fixed samples were stained with H&E and MASSON stains, and the OCT-embedded samples were stained with Oily Red O stains by the Centre for Pathology and PDX Efficacy Evaluation of China Pharmaceutical University. Individual pathological sections were observed using a fluorescence inverted microscope (bright field, DMI3000B, Leica, Wetzlar, Germany); the photographic field of view (200×) was selected along with the machine, and the section results were analyzed by Image J 1.53t.

### 4.4. Serum Biochemical Analysis

Serum ALT and AST activities were measured using commercial kits according to the manufacturer’s instructions (BC1555 for ALT and BC1565 for AST, Solarbio, Beijing, China).

### 4.5. Triglyceride and Triglyceride Measurement

Liver triglyceride (TG) and free fatty acid (NEFA) were tested strictly according to the instructions of TG Assay Kit and NEFA Assay Kit (A110-1-1 for TG and A042-2-1 for NEFA, Nanjing built).

### 4.6. Measurement of Mitochondrial Membrane Potential

Mitochondrial membrane potential was measured using the JC-1 Mitochondrial Membrane Potential Assay Kit (C2006, Beyotime, Shanghai, China). After washing twice, 200 μL of 1× JC-1 staining buffer was added to each well, and the fluorescence values of JC-1 monomer (excitation light 490 nm; emission light 530 nm) and JC-1 polymer (excitation light 525 nm; emission light 590 nm) were read by an enzyme marker. Mitochondrial membrane potential changes were observed by live cell imager (GFP, RFP).

### 4.7. BODIPY Staining

After 24 h of drug treatment of HepG2 cells, the cells were incubated with BODIPY working solution (BODIPY:PBS = 1:1000) for 25 min and washed three times with PBS, and 200 μL of PBS was added to each well; the accumulation of lipid droplets in the cells was observed by a live-cell imager (GFP), and the BODIPY staining images were analyzed by Image J 1.53t. The relative content of lipid droplets in the livers of the various groups was calculated.

### 4.8. Western Blot

Total proteins were extracted with RIPA lysate containing protease inhibitors, separated by SDS-PAGE and transferred to polyvinylidene difluoride membranes. And protein expression was detected using corresponding antibodies. The following antibodies were used: anti-alpha smooth muscle actin (#Ab124964; rabbit monoclonal antibody; Abcam (Cambridge, UK); 1:10,000 dilution), anti-GAPDH (#Ab181602; rabbit monoclonal antibody; Abcam; 1:10,000 dilution), anti-PPAR alpha (#Ab126285; rabbit polyclonal antibody; Abcam; 1:500 dilution), anti-NDUFS2 (#Ab192022; rabbit monoclonal antibody; Abcam; 1:10,000 dilution).

### 4.9. RNA Extraction and Quantitative RT-PCR Analysis

Total RNA was extracted from cells or tissues using Trizol (9108, TaKaRa, Tokyo, Japan) reagent, and cDNA was obtained using HiScript III RT SuperMix for qPCR (R323-01, Novozymes, Nanjing, China). cDNA was extracted from cells or tissues by ChamQ SYBR qPCR Master Mix (Q311-02, Novozymes, Nanjing, China) in a Step One ABI Real-Time PCR System for real-time quantitative PCR. The qPCR program was pre-denaturation: 95 °C, 90 s; denaturation: 95 °C, 10 s, annealing: 60 °C, 30 s, extension: 72 °C, 30 s, repeat 40 cycles. Finally, the melting curve was analyzed at 65~95 °C (0.5 °C/5 s). The qPCR system was 15 μL, and the specific proportion of each reagent is shown in Supplementary Table S1. The primers used are listed in Supplementary Table S2.

### 4.10. Cell Transfection with siRNA

Transfection of siRNA with Lipofectamine™ RNAiMAX Reagent (13778150, Invitrogen, Carlsbad, CA, USA) and NDUFS2 Gene siRNA Transfection Reagent. The siRNA sequences of the human NDUFS2 gene were 5′-GGAGAAAUCACACGUUUGUTT-3′ and 5′-ACAAACGUGUGAUUUCUCCTT-3′ (SiNDUFS2-969); 5′-GGCUUUAUGGAUGACAUUUUATT-3′ and 5′- UAAAUGUCAUCCAUAAGCCTT-3′ (SiNDUFS2-1162); 5′-CACUUGAUUCAUCACUUUAATT-3′ and 5′-UAAAUGUCAUCCAUAAGCCTT-3′ (SiNDUFS2-1576).

### 4.11. Liquid Chromatography-Tandem Mass Spectrometry (LC-MS/MS)

Processed liver and serum samples were detected using an AB SCIEX 5600 Q-TOF mass spectrometer (SCIEX, Framingham, MA, USA). Metabolomics chromatographic conditions—chromatographic column: Waters^®^ XBridge Amide 3.5 μm, 4.6 × 100 mm Colunm (Waters, Milford, MA, USA). Mobile phase and aqueous phase (A phase) ultrapure water: acetonitrile = 19:1 (*V*/*V*), mixing, adding 1 mL of ammonium acetate (5 mmoL/mL) and 670 μL of ammonia to adjust pH, shaking again, sonication for 5 min to remove air bubbles; organic phase (B phase) acetonitrile. Sonication for 5 min to remove air bubbles; the cap was loosened during sonication, and the organic phase (B phase) was acetonitrile. The temperature of the column oven was 40 °C; the flow rate was 0.4 mL/min; and the injection volume was 10 μL. Proteomic chromatography conditions—LC system: SCIEX M5 Microflow LC. Trap column: Chrom XP Eksigent, C18, 75 μm × 2 cm, 3 μm, 120 Å. Analytical column: Chrom XP Eksigent, C18, 300 μm × 2 cm, 3 μm, 120 Å. The analytical column: Chrom XP Eksigent, C18, 300 μm × 2 cm, 3 μm, 120 Å. Analytical column: Chrom XP Eksigent, C18, 300 μm × 15 cm, 3 μm, 120 Å. Mobile phases: G1 aqueous phase (phase A): water containing 0.1% formic acid. G1 organic phase (phase B): acetonitrile containing 0.1% formic acid. G2 aqueous phase (phase A): water containing 10% acetonitrile. G2 organic phase (phase B): acetonitrile containing 10% water. Metabolomics was based on the metabolomics database of the Key Laboratory of Pharmacokinetics, China Pharmaceutical University, and the metabolites were analyzed on MultiQuant 3.0.3 software to obtain the peak areas of each metabolite. For proteomics, the peak area of each sample was extracted by using the SWATH Acquisition module in PeakView software (SCIEX, Framingham, MA, USA, version 2.2).

### 4.12. Statistical Analysis

All data are expressed as mean (mean) ± standard error of the mean (SEM). One-way ANOVA (one-way analysis of variance) was used, followed by Tukey’s post hoc test for multiple group comparisons. Statistical analyses and histograms were performed using GraphPad Prism version 8.2.1 (GraphPad, La Jolla, CA, USA). All data are expressed as mean ± SEM, and the data are statistically significant when *p* < 0.05.

## 5. Conclusions

In summary, this study demonstrates that NGR1 exerts significant therapeutic effects against MASH by attenuating hepatic lipid accumulation, inflammatory responses, and fibrotic progression in CDAHFD-induced MASH mice. Mechanistically, we identify that NGR1 ameliorates MASH pathology primarily through restoration of NDUFS2 expression, which in turn improves mitochondrial function, enhances oxidative phosphorylation, and promotes fatty acid β-oxidation. These findings position NGR1 as a promising natural candidate for MASH therapy and highlight NDUFS2 as a potential key target for mitochondrial-targeted interventions in MASH. Future studies will further elucidate the upstream molecular mechanisms by which NGR1 regulates NDUFS2 expression and validates the central role of NDUFS2 in mediating the therapeutic effects of NGR1, thereby providing a theoretical foundation and practical strategy for the clinical treatment of MASH.

## Data Availability

The original contributions presented in this study are included in the article. Further inquiries can be directed to the corresponding authors.

## Supplementary Materials

The following supporting information can be downloaded at: https://www.mdpi.com/article/10.3390/ph19040524/s1, Figure S1: Body weight and food intake of mice fed with HFD.; Table S1: The system of quantitative real-time polymerase chain reaction.; Table S2: Human and mouse primer sequences.
