# Supplementary material for: NGR1 Ameliorates Hepatocyte Steatosis and Mitochondrial Dysfunction Associated with the Restoration of NDUFS2
Source: Pharmaceuticals (Basel). 2026 Mar 24;19(4):524. doi: 10.3390/ph19040524 (PMC13118776; doi:10.3390/ph19040524)
Supplement: Supplementary file 1 [file pharmaceuticals-19-00524-s001.zip › pharmaceuticals-4200788-supplementary.pdf]

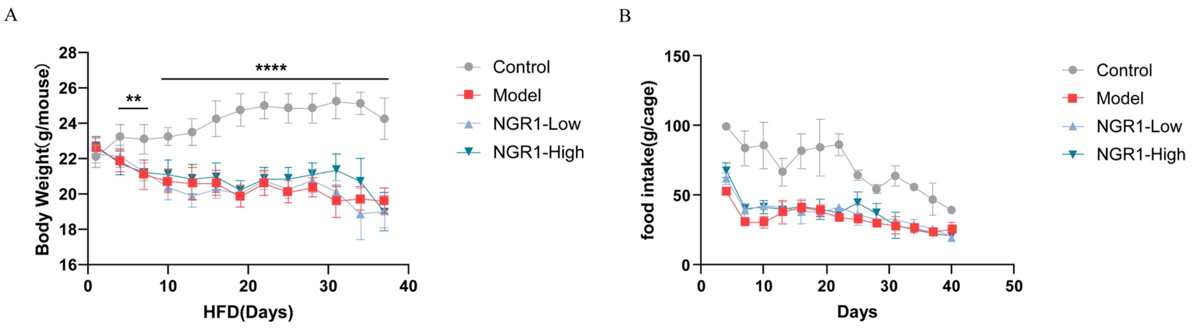

Figure S1 Body weight and food intake of mice fed with HFD. (A) Body weight of mice induced with HFD. (B) Daily food intake per cage was calculated from the change in food weekly weights. Data represented as mean ± SEM. Two-tailed unpaired Student's t test. **P* < 0.05, ***P* < 0.01, ****P* < 0.001. no statistical significance.

Table S1. The system of quantitative real-time polymerase chain reaction.

**Reagent name**	**Volume（μL）**
SYBR qPCR Master Mix	7.5
DEPC water（DNase、RNase free）	4.5
Forward primer	1
Reverse primer	1
cDNA	1

Table S2. Human and mouse primer sequences.

Gene	Forward primer	Reverse primer
Mouse-*Acta2*	CAGCCATCTTTCATTGGGATGGAG	AATGCCTGGGTACATGGTGG
Mouse-*Cd36*	AGATGACGTGGCAAAGAACAG	CCTTGGCTAGATAACGAACTCTG
Mouse-*Col1a1*	GAACTGGACTGTCCCAACCC	TTGGGTCCCTCGACTCCTAC
Mouse-*Gapdh*	AGGTCGGTGTGAACGGATTTG	GGGGTCGTTGATGGCAACA
Mouse-*Gpat1*	GCTGGCTGGCAGGAATCAT	GTCTGAGCCACCTCGGACAT
Mouse-*Il-1β*	TGTCTTGGCCGAGGACTAAG	TGGGCTGGACTGTTTCTAATGC
Mouse-*Tgf-β*	GCCCGAAGCGGACTACTATG	ATAGATGGCGTTGTTGCGGT

Mouse-*Tnf-α*	ACGGCATGGATCTCAAA	AGATAGCAAATCGGCTGAC
Human-*ACTB*	GCGTGACATTAAGGAGAAG	GAAGGAAGGCTGGAAGAG
Human-*CPT1α*	ATGCGCTACTCCCTGAAAGTG	GTGGCACGACTCATCTTGC
Human-*NDUFS2*	CTCCACCTAAGCGAGCAGAG	ACACCCCAAACTCTCCCTTG
Human-*PPGC-1α*	GCTTTCTGGGTGGACTCAAGT	GAGGGCAATCCGTCTTCATCC
Human-*PPARα*	TTCGCAATCCATCGGCGAG	CCACAGGATAAGTCACCGAGG
Human-GAPDH	GGAGCGAGATCCCTCCAAAAT	GGCTGTTGTCATACTTCTCATGG
Human-*SIRT1*	AAGTTGACTGTGAAGCTGTACG	TGCTACTGGTCTTACTTTGAGGG
Mouse-*Acadl*	TGCCCTATATTGCGAATTACGG	CTATGGCACCGATACACTTGC
Mouse-*Acadm*	AACACAACACTCGAAAGCGG	TTCTGCTGTTCCGTCAACTCA
Mouse-*Acadvl*	ACTACTGTGCTTCAGGGACAA	GCAAAGGACTTCGATTCTGCC
Mouse-*Cpt1α*	TGGCATCATCACTGGTGTGTT	GTCTAGGGTCCGATTGATCTTTG
Mouse-*Cpt2*	CCTGCTCGCTCAGGATAAACA	GTGTCTTCAGAAACCGCACTG
Mouse-*Ndufs2*	TCGTGCTGGAACTGAGTGGA	GGCCTGTTCATTACACATCATGG
Mouse-*Pgc-1α*	TATGGAGTGACATAGAGTGTGCT	GTCGCTACACCACTTCAATCC
Mouse-*Pparα*	TTTCGGCGAACTATTCGGCTG	GGCATTTGTTCCGGTTCTTCTT
Mouse-*Sirt1*	TGATTGGCACCGATCCTCG	CCACAGCGTCATATCATCCAG
